# Poly[dichloridobis[μ-1-(4-pyridyl­meth­yl)-1,2,4-triazole]cadmium(II)]

**DOI:** 10.1107/S1600536810036251

**Published:** 2010-09-15

**Authors:** Jian Wang, Zhu-Lai Li, Xiu-Zhi Xu, Wen-Jin Yan, Hui-Li Chi

**Affiliations:** a Department of Medicinal Chemistry, School of Pharmacy, Fujian Medical University, Fuzhou, Fujian 350004, People’s Republic of China

## Abstract

In the title coordination polymer, [CdCl_2_(C_8_H_8_N_4_)_2_]_*n*_, the Cd^II^ atom, lying on an inversion center, is coordinated by two Cl atoms and two triazole N atoms and two pyridyl N atoms from four 1-(4-pyridyl­meth­yl)-1,2,4-triazole (pmta) ligands in a distorted *trans*-CdCl_2_N_4_ octa­hedral arrangement. The bridg­ing pmta ligands, with a dihedral angle between the triazole and pyridyl rings of 71.86 (8)°, link the Cd atoms into a 4^4^ sheet parallel to (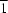
02). π–π inter­actions between the triazole rings [centroid–centroid distance = 3.428 (2) Å] connect the sheets.

## Related literature

For our previous studies on the design and synthesis of some unsymmetric flexible ligands, see: Huang *et al.* (2006 ▶); Liu *et al.* (2005 ▶). For related structures, see: Li *et al.* (2009 ▶); Wang *et al.* (2008 ▶).
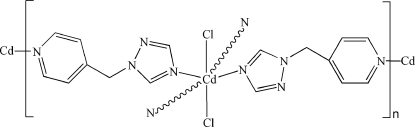


## Experimental

### 

#### Crystal data

[CdCl_2_(C_8_H_8_N_4_)_2_]
*M* *_r_* = 503.67
Monoclinic, 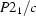

*a* = 7.5795 (5) Å
*b* = 16.9491 (10) Å
*c* = 8.2215 (5) Å
β = 113.325 (3)°
*V* = 969.86 (10) Å^3^
*Z* = 2
Mo *K*α radiation
μ = 1.42 mm^−1^
*T* = 293 K
0.20 × 0.18 × 0.04 mm

#### Data collection

Rigaku Mercury CCD diffractometer
Absorption correction: multi-scan (*CrystalClear*; Rigaku, 2007 ▶) *T*_min_ = 0.841, *T*_max_ = 1.000
6994 measured reflections
2214 independent reflections
2079 reflections with *I* > 2σ(*I*)
*R*_int_ = 0.016

#### Refinement

*R*[*F* ^2^ > 2σ(*F* ^2^)] = 0.022
*wR*(*F* ^2^) = 0.048
*S* = 1.01
2214 reflections
124 parameters
H-atom parameters constrained
Δρ_max_ = 0.78 e Å^−3^
Δρ_min_ = −0.23 e Å^−3^

### 

Data collection: *CrystalClear* (Rigaku, 2007 ▶); cell refinement: *CrystalClear*; data reduction: *CrystalClear*; program(s) used to solve structure: *SHELXS97* (Sheldrick, 2008 ▶); program(s) used to refine structure: *SHELXL97* (Sheldrick, 2008 ▶); molecular graphics: *SHELXTL* (Sheldrick, 2008 ▶) and *DIAMOND* (Brandenburg, 1999 ▶); software used to prepare material for publication: *SHELXTL*.

## Supplementary Material

Crystal structure: contains datablocks I, global. DOI: 10.1107/S1600536810036251/hy2349sup1.cif

Structure factors: contains datablocks I. DOI: 10.1107/S1600536810036251/hy2349Isup2.hkl

Additional supplementary materials:  crystallographic information; 3D view; checkCIF report

## Figures and Tables

**Table 1:** Selected bond lengths (Å)

Cd1—N3	2.3531 (16)
Cd1—N4^i^	2.4183 (16)
Cd1—Cl1	2.5842 (5)

Symmetry code: (i) 
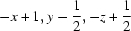
.

**Fig. 1.**
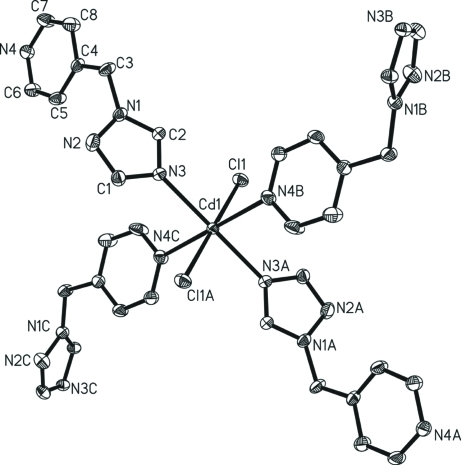
The structure of the title compound, showing 30% probability displacement ellipsoids. H atoms have been omitted for clarity. [Symmetry codes: (A) -x, -y+1, -z; (B) -x+1, y-1/2, -z+1/2; (C) x-1, -y+3/2, z-1/2.]

**Fig. 2.**
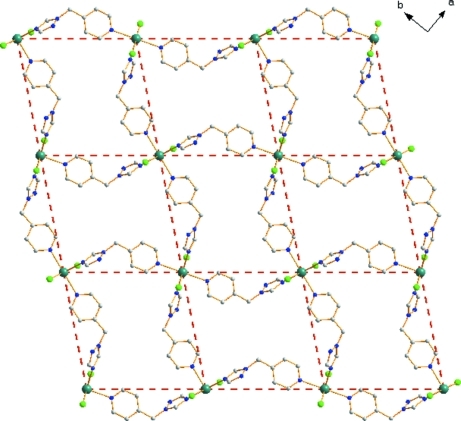
The two-dimensional structure of the title compound, constructed of rhombus-shaped grids.

## supplementary crystallographic information

### Comment

Recently, our group has focused on the design and synthesis of some unsymmetric flexible ligands (Huang *et al.*, 2006; Liu *et al.*, 2005), and we have got a heterocyclic ligand 1-(4-pyridylmethyl)-1,2,4-triazole (pmta). In order to explore the architectural styles and coordination chemistry of this kind of ligands, we selected cadmium chloride as representative subject for stereoregular coordination. Among our attempts, the title compound, a new coordination polymer, was obtained as crystals suitable for single-crystal X-ray analysis.

The title compound is isomorphic to the complex we have reported (Li *et al.*, 2009; Wang *et al.*, 2008). The crystallographic analysis reveals that the asymmetric unit contains one Cd^II^ atom lying on an inversion center, one Cl anion and one bridging pmta ligand, as shown in Fig. 1. The Cd^II^ atom lies in an octahedral [CdCl_2_N_4_] environment, with the axial positions occupied by two Cl atoms and the equatorial positions occupied by two *trans* triazole N atoms and two *trans* pyridyl N atoms, which belong to four different pmta ligands. The bond angles about Cd1 atom range from 85.82 (6) to 94.18 (6)° and deviate slightly from those of a perfect octahedron. Due to the existence of the –CH_2_– spacer between the triazole and pyridyl rings with a dihedral angle of 71.86 (8)°, sufficient flexibility makes it possible for pmta to be twisted to meet the requirment of coordination geometries of the Cd center.

As shown in Fig. 2, the title compound exhibits a two-dimensional rhombohedral sheet containing 36-membered sandglass rings. The *sp*^3^ configuration of C3 forces the pmta ligand to be non-linear, generating the non-linear grid sides and thereby the sandglass grids. Every complementary four [Cd_4_(pmta)_4_] grids are joined together by sharing the Cd apices, giving a 4^4^ topology with a side length of 11.022 Å and diagonal measurements of 14.096 and 16.949 Å.

### Experimental

A solution of pmta (0.021 g, 0.10 mmol) in MeOH (5 ml) was carefully layered on a solution of CdCl_2_.2.5H_2_O (0.023 g, 0.10 mmol) in H_2_O (5 ml). Diffusion between the two phases over a period of two weeks produced colorless block crystals.

### Refinement

H atoms were positioned geometrically and refined as riding atoms, with C—H = 0.93 (CH) and 0.97 (CH_2_) Å and with *U*_iso_(H) = 1.2*U*_eq_(C).

### Crystal data

**Table:**

[CdCl_2_(C_8_H_8_N_4_)_2_]	*F*(000) = 500
*M**_r_* = 503.67	*D*_x_ = 1.725 Mg m^−^^3^
Monoclinic, *P*2_1_/*c*	Mo *K*α radiation, λ = 0.71073 Å
Hall symbol: -P 2ybc	Cell parameters from 2546 reflections
*a* = 7.5795 (5) Å	θ = 2.7–27.5°
*b* = 16.9491 (10) Å	µ = 1.42 mm^−^^1^
*c* = 8.2215 (5) Å	*T* = 293 K
β = 113.325 (3)°	Prism, colorless
*V* = 969.86 (10) Å^3^	0.20 × 0.18 × 0.04 mm
*Z* = 2	

### Data collection

**Table:**

Rigaku Mercury CCD diffractometer	2214 independent reflections
Radiation source: fine-focus sealed tube	2079 reflections with *I* > 2σ(*I*)
graphite	*R*_int_ = 0.016
ω scans	θ_max_ = 27.5°, θ_min_ = 3.0°
Absorption correction: multi-scan (*CrystalClear*; Rigaku, 2007)	*h* = −9→9
*T*_min_ = 0.841, *T*_max_ = 1.000	*k* = −15→22
6994 measured reflections	*l* = −9→10

### Refinement

**Table:**

Refinement on *F*^2^	Primary atom site location: structure-invariant direct methods
Least-squares matrix: full	Secondary atom site location: difference Fourier map
*R*[*F*^2^ > 2σ(*F*^2^)] = 0.022	Hydrogen site location: inferred from neighbouring sites
*wR*(*F*^2^) = 0.048	H-atom parameters constrained
*S* = 1.00	*w* = 1/[σ^2^(*F*_o_^2^) + (0.016*P*)^2^ + 0.9435*P*] where *P* = (*F*_o_^2^ + 2*F*_c_^2^)/3
2214 reflections	(Δ/σ)_max_ < 0.001
124 parameters	Δρ_max_ = 0.78 e Å^−^^3^
0 restraints	Δρ_min_ = −0.23 e Å^−^^3^

### Fractional atomic coordinates and isotropic or equivalent isotropic displacement parameters (Å^2^)

**Table:**

	*x*	*y*	*z*	*U*_iso_*/*U*_eq_	
Cd1	0.0000	0.5000	0.0000	0.02465 (6)	
Cl1	0.18617 (7)	0.53526 (3)	−0.19411 (7)	0.03766 (12)	
N2	0.4333 (3)	0.60582 (12)	0.5065 (2)	0.0416 (4)	
C2	0.4379 (3)	0.56302 (12)	0.2553 (3)	0.0309 (4)	
H2	0.4822	0.5515	0.1674	0.037*	
C3	0.7421 (3)	0.62024 (12)	0.4780 (3)	0.0387 (5)	
H3A	0.8033	0.5921	0.4116	0.046*	
H3B	0.8016	0.6027	0.6000	0.046*	
C4	0.7814 (3)	0.70736 (11)	0.4718 (3)	0.0315 (4)	
C5	0.6464 (3)	0.76634 (13)	0.4358 (3)	0.0404 (5)	
H5	0.5187	0.7540	0.4106	0.048*	
C6	0.7022 (3)	0.84449 (12)	0.4373 (3)	0.0385 (5)	
H6	0.6094	0.8836	0.4141	0.046*	
C7	1.0109 (3)	0.80837 (14)	0.5028 (4)	0.0501 (6)	
H7	1.1368	0.8221	0.5239	0.060*	
C8	0.9684 (3)	0.72969 (13)	0.5070 (4)	0.0496 (6)	
H8	1.0646	0.6919	0.5332	0.060*	
N3	0.2643 (2)	0.54659 (10)	0.2469 (2)	0.0306 (4)	
C1	0.2694 (3)	0.57354 (12)	0.4037 (3)	0.0356 (4)	
H1	0.1653	0.5695	0.4360	0.043*	
N1	0.5399 (2)	0.59858 (9)	0.4081 (2)	0.0309 (4)	
N4	0.8817 (3)	0.86600 (10)	0.4702 (2)	0.0351 (4)	

### Atomic displacement parameters (Å^2^)

**Table:**

	*U*^11^	*U*^22^	*U*^33^	*U*^12^	*U*^13^	*U*^23^
Cd1	0.02100 (10)	0.02176 (10)	0.03014 (10)	0.00065 (7)	0.00902 (7)	−0.00232 (7)
Cl1	0.0338 (3)	0.0460 (3)	0.0388 (3)	0.0022 (2)	0.0204 (2)	0.0020 (2)
N2	0.0476 (11)	0.0420 (11)	0.0385 (10)	−0.0133 (9)	0.0205 (9)	−0.0113 (8)
C2	0.0264 (9)	0.0316 (10)	0.0330 (10)	−0.0031 (8)	0.0097 (8)	−0.0057 (8)
C3	0.0261 (10)	0.0265 (10)	0.0511 (13)	−0.0061 (8)	0.0022 (9)	−0.0011 (9)
C4	0.0307 (10)	0.0257 (9)	0.0331 (10)	−0.0049 (8)	0.0073 (8)	−0.0019 (8)
C5	0.0274 (10)	0.0317 (11)	0.0579 (14)	−0.0051 (8)	0.0125 (10)	0.0022 (10)
C6	0.0320 (11)	0.0276 (10)	0.0534 (13)	0.0007 (8)	0.0141 (10)	0.0039 (9)
C7	0.0315 (11)	0.0284 (11)	0.094 (2)	−0.0065 (9)	0.0285 (12)	−0.0064 (12)
C8	0.0338 (12)	0.0250 (11)	0.0862 (19)	−0.0009 (9)	0.0197 (12)	−0.0064 (11)
N3	0.0246 (8)	0.0318 (9)	0.0334 (8)	−0.0030 (7)	0.0095 (7)	−0.0038 (7)
C1	0.0390 (11)	0.0336 (11)	0.0387 (11)	−0.0064 (9)	0.0202 (9)	−0.0052 (9)
N1	0.0283 (8)	0.0235 (8)	0.0365 (9)	−0.0051 (6)	0.0082 (7)	−0.0010 (7)
N4	0.0345 (9)	0.0246 (8)	0.0479 (10)	−0.0036 (7)	0.0180 (8)	−0.0005 (7)

### Geometric parameters (Å, °)

**Table:**

Cd1—N3	2.3531 (16)	C4—C8	1.383 (3)
Cd1—N4^i^	2.4183 (16)	C5—C6	1.389 (3)
Cd1—Cl1	2.5842 (5)	C5—H5	0.9300
N2—C1	1.313 (3)	C6—N4	1.329 (3)
N2—N1	1.357 (3)	C6—H6	0.9300
C2—N3	1.320 (2)	C7—N4	1.333 (3)
C2—N1	1.330 (2)	C7—C8	1.375 (3)
C2—H2	0.9300	C7—H7	0.9300
C3—N1	1.454 (2)	C8—H8	0.9300
C3—C4	1.511 (3)	N3—C1	1.353 (3)
C3—H3A	0.9700	C1—H1	0.9300
C3—H3B	0.9700	N4—Cd1^ii^	2.4183 (16)
C4—C5	1.376 (3)		
			
N3^iii^—Cd1—N3	180.0	C5—C4—C3	125.27 (19)
N3^iii^—Cd1—N4^i^	85.82 (6)	C8—C4—C3	117.38 (19)
N3—Cd1—N4^i^	94.18 (6)	C4—C5—C6	119.5 (2)
N3^iii^—Cd1—N4^iv^	94.18 (6)	C4—C5—H5	120.2
N3—Cd1—N4^iv^	85.82 (6)	C6—C5—H5	120.2
N4^i^—Cd1—N4^iv^	180.00 (10)	N4—C6—C5	123.2 (2)
N3^iii^—Cd1—Cl1	91.79 (4)	N4—C6—H6	118.4
N3—Cd1—Cl1	88.21 (4)	C5—C6—H6	118.4
N4^i^—Cd1—Cl1	90.49 (5)	N4—C7—C8	123.7 (2)
N4^iv^—Cd1—Cl1	89.51 (5)	N4—C7—H7	118.1
N3^iii^—Cd1—Cl1^iii^	88.21 (4)	C8—C7—H7	118.1
N3—Cd1—Cl1^iii^	91.79 (4)	C7—C8—C4	119.4 (2)
N4^i^—Cd1—Cl1^iii^	89.51 (5)	C7—C8—H8	120.3
N4^iv^—Cd1—Cl1^iii^	90.49 (5)	C4—C8—H8	120.3
Cl1—Cd1—Cl1^iii^	180.0	C2—N3—C1	103.13 (16)
C1—N2—N1	102.33 (17)	C2—N3—Cd1	127.31 (13)
N3—C2—N1	109.85 (18)	C1—N3—Cd1	128.94 (14)
N3—C2—H2	125.1	N2—C1—N3	114.62 (19)
N1—C2—H2	125.1	N2—C1—H1	122.7
N1—C3—C4	115.10 (17)	N3—C1—H1	122.7
N1—C3—H3A	108.5	C2—N1—N2	110.07 (17)
C4—C3—H3A	108.5	C2—N1—C3	127.96 (19)
N1—C3—H3B	108.5	N2—N1—C3	121.64 (18)
C4—C3—H3B	108.5	C6—N4—C7	116.82 (18)
H3A—C3—H3B	107.5	C6—N4—Cd1^ii^	125.95 (14)
C5—C4—C8	117.34 (19)	C7—N4—Cd1^ii^	117.03 (14)

Symmetry codes: (i) −*x*+1, *y*−1/2, −*z*+1/2; (ii) −*x*+1, *y*+1/2, −*z*+1/2; (iii) −*x*, −*y*+1, −*z*; (iv) *x*−1, −*y*+3/2, *z*−1/2.

## References

[bb1] Brandenburg, K. (1999). *DIAMOND* Crystal Impact GbR, Bonn, Germany.

[bb2] Huang, M., Liu, P., Chen, Y., Wang, J. & Liu, Z. (2006). *J. Mol. Struct.* **788**, 211–217.

[bb3] Li, Z.-L., Wang, J., Xu, X.-Z. & Ye, X. (2009). *Acta Cryst.* E**65**, m340. 10.1107/S160053680900645X PMC2968461 21582107

[bb4] Liu, Z., Liu, P., Chen, Y., Wang, J. & Huang, M. (2005). *Inorg. Chem. Commun.* **8**, 212–215.

[bb5] Rigaku (2007). *CrystalClear* Rigaku Corporation, Tokyo, Japan.

[bb6] Sheldrick, G. M. (2008). *Acta Cryst.* A**64**, 112–122. 10.1107/S0108767307043930 18156677

[bb7] Wang, J., Huang, M., Liu, P. & Cheng, W. (2008). *J. Mol. Struct.* **875**, 22–26.

